# Supplementary material for: Physiological and Transcriptome Analyses of Photosynthesis in Three Mulberry Cultivars within Two Propagation Methods (Cutting and Grafting) under Waterlogging Stress
Source: Plants (Basel). 2023 May 23;12(11):2066. doi: 10.3390/plants12112066 (PMC10255098; doi:10.3390/plants12112066)
Supplement: Supplementary file 1 [file plants-12-02066-s001.zip › plants-2368455-supplementary.pdf]

Article

Physiological and Transcriptome Analyses of Photosynthesis in Three Mulberry Cultivars within Two Propagation Methods (Cutting and Grafting) under Waterlogging Stress

Yong Li [1], Jin Huang [1], Cui Yu [1], Rongli Mo [1], Zhixian Zhu [1], Zhaoxia Dong [1], Xingming Hu [1], Chuxiong Zhuang [2,*] and Wen Deng [1,*]

[1] Cash Crops Research Institute, Hubei Academy of Agricultural Sciences, Wuhan 430064, China
[2] College of Life Sciences, South China Agricultural University, Guangzhou 510642, China
* Correspondence: zhuangcx@scau.edu.cn (C.Z.); dengwen@hbaas.com (W.D.)

Table S2. The primer sequences used for RT-qPCR.

Seq ID	gene name	Primer sequences
L-2930F	*LHCB2*	ATCCAACAATCGGCATTC
L-2930R		CTCCTAACAGTTCTCCTCAT
L-5857F	*LHCB5*	TGAAGGTGAAGGAGATTAAGA
L-5857R		CAATGACAGTGAGCAAGTT
L-8660F	*LHCB4*	CTCCATCACCACACTCAT
L-8660R		ATCTCAGCCAGTTGAAGG
L-5037F	*petC*	ACAATGGCTTCCTCAACT
L-5037R		TTCCCTTTCCCTTTACTACC
L-9768F	*LHCA5*	ATATCAAGAATGCTCATCACTG
L-9768R		ATGCTCCACAAGATTATCAAC
L-6592F	*LHCA1*	CTCTCCATTGCCTTCGTA
L-6592R		TTGTATTCCTTGAACTTCTCTG
L-9752F	*FEDA*	TGGTTGGTTGGATGGATTA
L-9752R		GAAGTTGTATTAGTGTGGTTGA
L-4966F	*LHCA2*	CTGAGACCTTGAGATGGAA
L-4966R		GTAGTTGTGTCTGTGAAGTATT
L-7447F	*petE*	GCTACGCCTTCTACTGTT
L-7447R		TTCCACCACTTAACTATAATATCG
L-8965F	*psb28*	TGTGGTTGTCGTTGTTAC
L-8965R		TGGTGAAGATGGTGAGAG

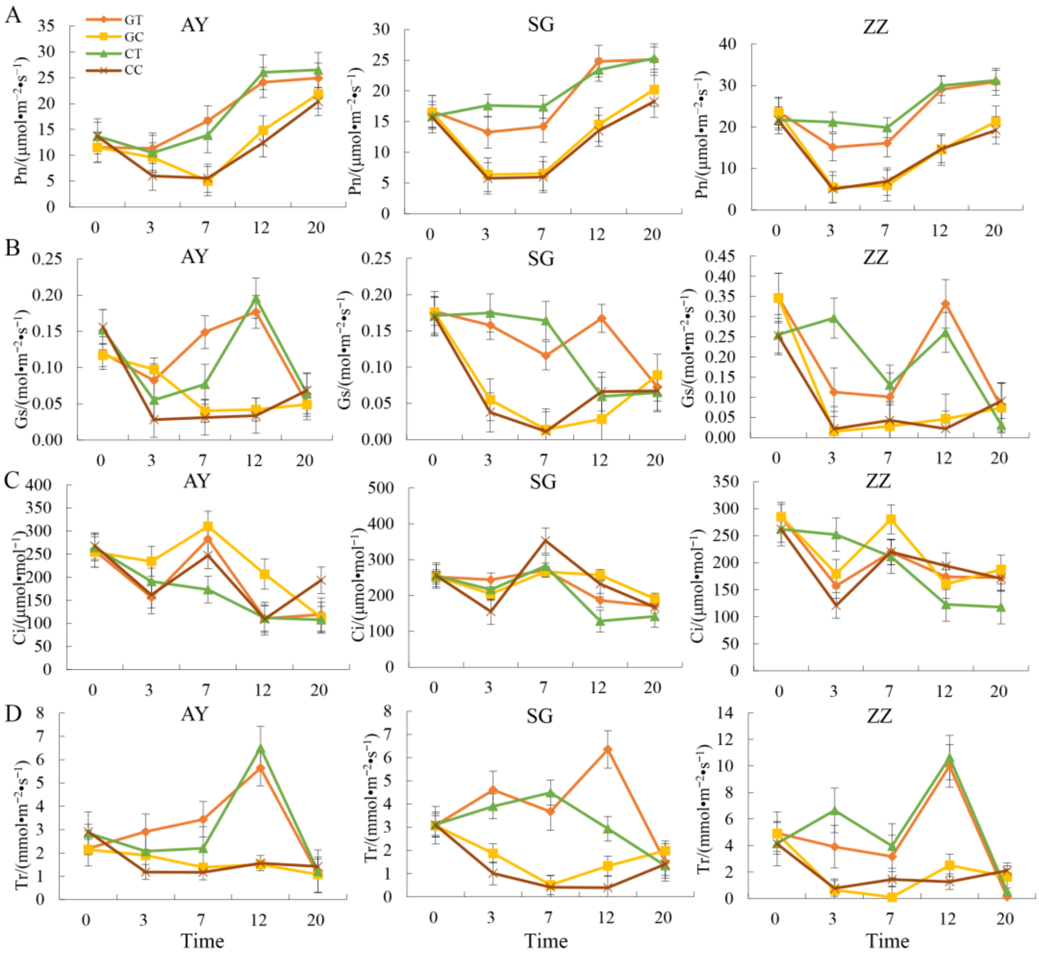

Figure S1. Dynamics of the photosynthetic characters, including *P*n **(A)**, *G*s **(B)**, *C*i **(C)**, and *T*r **(D)**, in three mulberry cultivars after waterlogging treatments. CC, CT, GC and GT in the legend indicate cut mulberry under control and waterlogging treatment, grafted mulberry under control and waterlogging treatment, respectively.

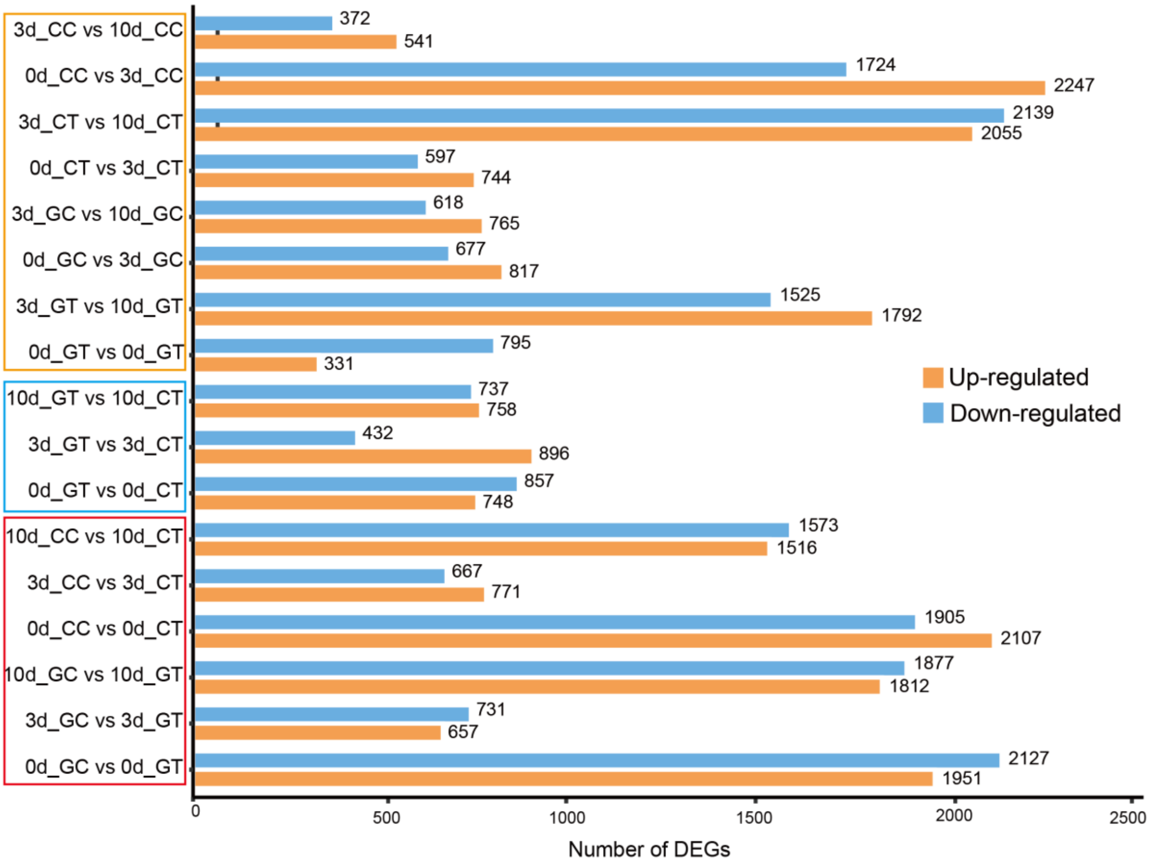

Figure S2. The number of DEGs identified with 17 pairwise comparisons. Red frame including Control (C) vs. Waterlogging treatments (T) in D0, D3 and D10 for cutting (C) and grafting (G); bule frame including grafting vs. cutting under waterlogging; orange frame including dynamic changes of time between control vs. waterlogging condition in cutting and grafting. CC, CT, GC and GT in the legend indicate cut mulberry under control and waterlogging treatment, grafted mulberry under control and waterlogging treatment, respectively.

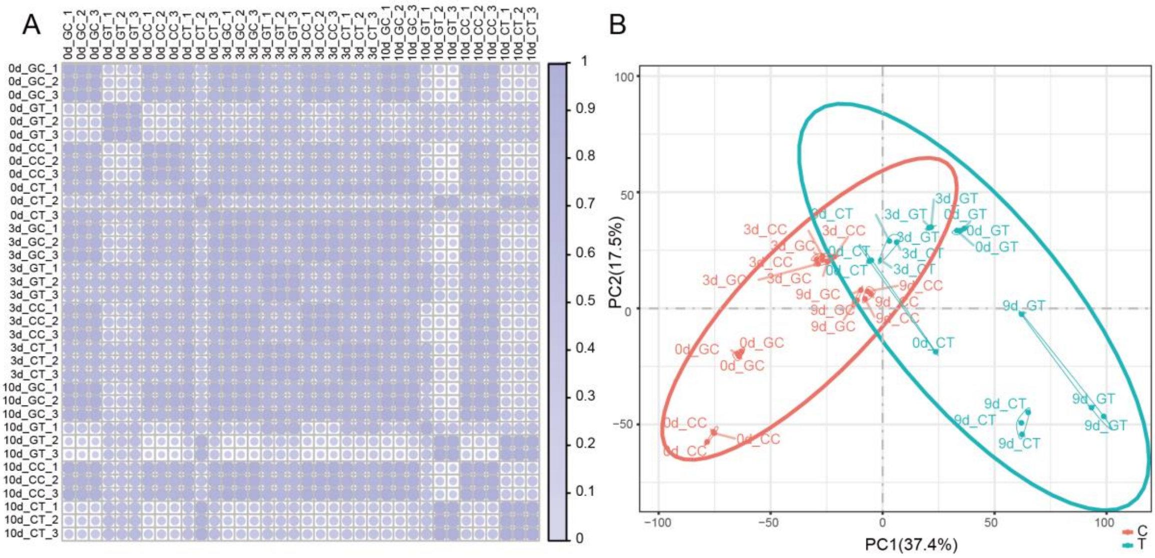

Figure S3. Pairwise Pearson correlation coefficient **(A)** and PCA analysis for 36 sample with FPKM **(B)**. The X axis represents PC1, and the Y axis represents PC2. Each sample has three biological duplicates and one plot represented a sample. CC, CT, GC and GT in the legend indicate cut mulberry under control and waterlogging treatment, grafted mulberry under control and waterlogging treatment, respectively.

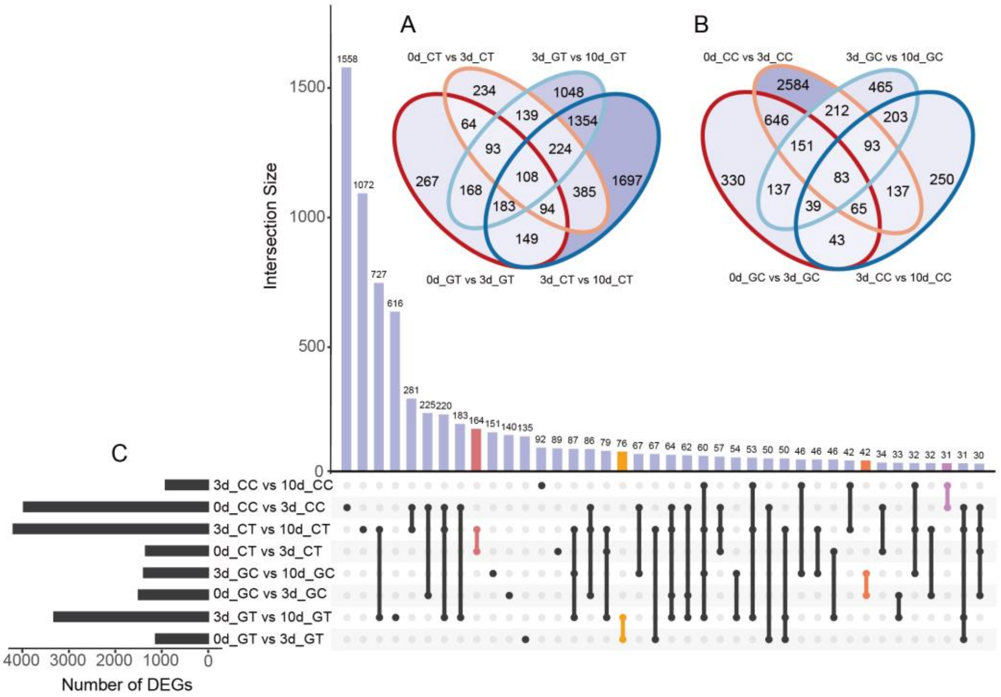

Figure S4. Venn diagram and UpSet indicated the number of differentially expressed genes (DEGs) per contrast; Divided into two groups including grafting and cutting under control **(A)** and waterlogging treatments **(B)** for dynamic change from D0 to D3, D3 to D10; UpSet summary for 8 pairwise comparisons (**C**). Numbers in intersections represent the number of DEGs shared in the intersection contrasts.
